# Construction of a ferroptosis-based prognostic model for breast cancer helps to discriminate high/low risk groups and treatment priority

**DOI:** 10.3389/fimmu.2023.1264206

**Published:** 2023-12-13

**Authors:** Liyong Zhang, Tingting Zhao, Xiujuan Wu, Hao Tian, Pingping Gao, Qingqiu Chen, Ceshi Chen, Yi Zhang, Shushu Wang, Xiaowei Qi, Na Sun

**Affiliations:** ^1^ Department of Breast and Thyroid Surgery, Southwest Hospital, Army Medical University, Chongqing, China; ^2^ Key Laboratory of Animal Models and Human Disease Mechanisms of the Chinese Academy of Sciences and Yunnan Province, Kunming Institute of Zoology, Chinese Academy of Sciences, Kunming, China

**Keywords:** ferroptosis, breast cancer, prognostic model, TCGA, GEO

## Abstract

**Introduction:**

Breast cancer is a common malignant tumor associated with high morbidity and mortality. The role of ferroptosis, a regulated form of cell death, in breast cancer development and prognosis remains unclear. This study aims to investigate the relationship between ferroptosis-related genes and breast cancer and develop a prognostic model.

**Methods:**

RNA-seq expression datasets and clinical samples of breast cancer patients were obtained from public databases. Immunity- and drug resistance-related data were integrated. A preliminary screening was performed, resulting in the identification of 73 candidate ferroptosis factors. Univariate Cox regression analysis was conducted to select 12 genes, followed by LASSO Cox regression analysis to construct a prognostic risk prediction model consisting of 10 ferroptosis-related genes. The model was further characterized by immune cell infiltration. The expression levels of ferroptosis-related genes were validated in human breast cancer cell lines, and immunohistochemical (IHC) analysis was conducted on cancer specimens to assess ferroptosis-related protein expression.

**Results:**

The study identified 10 ferroptosis-related genes that were significantly associated with breast cancer prognosis. The constructed prognostic risk prediction model showed potential for predicting the prognostic value of these genes. In addition, the infiltration of immune cells was observed to be a characteristic of the model. The expression levels of ferroptosis-related genes were confirmed in human breast cancer cell lines, and IHC analysis provided evidence of ferroptosis-related protein expression in cancer specimens.

**Discussion:**

This study provides a novel prognostic model for breast cancer, incorporating 10 ferroptosis-related genes. The model demonstrates the potential for predicting breast cancer prognosis and highlights the involvement of immune cell infiltration. The expression levels of ferroptosis-related genes and proteins further support the association between ferroptosis and breast cancer development.

## Introduction

It was predicted that in 2020, approximately 2.3 million new cases of breast cancer would occur and account for 11.7% of all new cancer cases (1). Due to the heterogeneity of breast cancer molecular subtypes, the current therapeutic effects, and patient prognosis are still unsatisfactory. Although major progress has been made in its treatment through surgery, radiation therapy, hormone therapy, targeted therapy, etc., the mortality rate of breast cancer is still high (2). Predicting the prognosis of patients and treatment outcome is important from the viewpoint of treatment management and improving survival. Currently, growing evidence suggests that induction of ferroptosis may improve the efficacy of tumor therapy (3–5). However, there was currently little research on ferroptosis-related signature genes in breast cancer patients.

Ferroptosis is a newly identified regulated cell death mechanism that is characterized by the destruction of intracellular redox balance and non-apoptotic pathways (6). In recent years, studies have suggested that ferroptosis may be triggered, for example, in the treatment of aggressive malignancies that are resistant to conventional therapy and have a poor prognosis (7). Various other studies have also suggested that ferroptosis plays an inhibitory role in tumor growth and progression, while the growth of chemotherapy-resistant tumors is inhibited by induction of ferroptosis (8, 9). With regard to breast cancer, it has been reported that erastin encapsulated by targeting exosomes induces ferroptosis in TNBC cells (10). Further, another study showed that estrogen receptor inhibits sulfasalazine-induced ferroptosis in breast cancer cells by inhibiting transferrin receptors (11). Another study found that *NCOA3* acts as a co-activator in synergy with *NR5A2* to prevent BETi-induced ferroptosis (12). Despite these findings, the association between ferroptosis-related signature genes and the prognosis of breast cancer patients remains unclear. Therefore, in the current study, we downloaded the RNA-Seq expression datasets and clinical samples datasets of breast cancer patients from The Cancer Genome Atlas (TGGA) datasets and constructed a prognostic polygenic model with ferroptosis-related signature genes. Further, we verified the predictive ability of the model using clinical data for breast cancer from the GEO dataset.

In recent years, there has been increasing research on the immune microenvironment, especially the infiltration of immune cells. According to reports, the immune microenvironment is critical for the development of breast cancer, but its specific mechanism is not yet clear (13). Therefore, determining the immune-related molecular mechanism of breast cancer tumor growth is very important for the treatment of breast cancer. In this present study, functional enrichment analysis of the identified ferroptosis-related DEGs was performed to explore potential immune-related mechanisms of breast cancer. Additionally, sensitivity to immunotherapy and chemotherapy was also analyzed.

## Materials and methods

### Cell lines and cultures

The human breast cancer cell lines MDA-KB-2, MDA-MB-231, T47D, and MDA-MB-453 were cultured in Dulbecco’s modified Eagle’s medium (DMEM) containing high glucose (4.5 g/L glucose) from HyClone, USA. The cell lines SK-BR-3, BT474, and ZR-75-1 were cultured in RPMI-1640 medium from HyClone. Both media were supplemented with 10% fetal bovine serum from Biochannel, Nanjing, China, as well as 100 U/mL penicillin and 100 mg/mL streptomycin. All cell lines were incubated at 37°C and 5% CO_2_ in a CO_2_ incubator (SANYO MCO-175, Japan).

### Patient tissue samples

A total of 7 tissue samples were collected, including the Tumor group (patients with breast cancer) and the Normal group (patients with fibroadenoma and cyclomastopathy) (**Table S1**) (Southwestern Hospital, Army Medical University, Chongqing, China). This study was approved by the Ethics Committee of the First Affiliated Hospital of the Army Medical University (KY2020055), and the study subjects signed an informed consent form.

### RNA extraction and qRT-PCR

Total RNA was isolated from breast cancer cell lines and normal breast cell lines using TRIzol Reagent from Servicebio, Wuhan, China. Approximately 1 μg of the total RNA was then reverse-transcribed into cDNA using the PrimeScript™ RT Reagent Kit with gDNA Eraser (Perfect Real Time) according to the instructions provided by the manufacturer, Takara, Dalian, China.

For quantitative real-time PCR (qRT-PCR), TB Green™ Premix Ex Taq II from TaKaRa was used. The qRT-PCR was performed on a CFX Connect™ Real-Time PCR Detection System from Bio-Rad, Hercules, CA, USA. The expression levels of TP63, SQLE, SLC7A5, SLC7A11, PTGS2, PROM2, MT3, IL33, ANO6, ALOX15B were normalized using β-Actin as the internal control. In this study, quantitative real-time PCR (qRT-PCR) was performed to analyze gene expression levels. The specific primer sequences used for amplifying the target genes were as follows:

TP63: TCGTCAGAACACACATGGTATCC (forward); GCTGTTGCCTGTACGTTTCAATT (reverse).

SQLE: AAGCTTCCTTCCTCCTTCATCAG(forward); CAACAGTCATTCCTCCACCAGTA (reverse).

SLC7A5: GGGAACATTGTGCTGGCATTATA(forward); CCAGGTGATAGTTCCCGAAGTC (reverse).

SLC7A11: CTTTCAAGGTGCCACTGTTCATC (forward); Reverse: ACGAAGCCAATCCCTGTACTAAA (reverse).

PTGS2: CTTCCTCCTGTGCCTGATGATTG(forward); CCCTCGCTTATGATCTGTCTTGA (reverse).

PROM2: AGCCTGAAAGTAGACACACAGAG (forward); CTCTGGATCTGAACGAGGAAGTC (reverse).

MT3: CTGCTGCTCTCCTCGACATG (forward); CTTTGCACACACAGTCCTTGG

IL33: ATGAATCAGGTGACGGTGTTGAT (forward); TCCACAGAGTGTTCCTTGTTGTT (reverse).

ANO6: TCCCACTCCATGATTGCAAATTC (forward); TAATAGCCCAGCCAAGCAAAGTA (reverse).

ALOX15B: CTGTCACTACCTCCCAAAGAACT (forward); AGAGAACTGAGGCTTCCCATTAA (reverse).

Data analysis was performed using the comparative Ct method (ΔΔCt).

### Immunohistochemistry and single-cell RNA-seq analysis

The protein expression of 10 ferroptosis-related genes involved in the signature, among TCGA-BRCA samples, was analyzed by IHC using the available scanned tumor staining from Human Protein Atlas (HPA) database (https://www.proteinatlas.org/). The information of IHC staining was determined and manually adjusted by experts from the HPA database, and ferroptosis-related genes IHC staining was defined and exhibited as high, medium, low staining or not detected. IHC analysis was employed to evaluate the expression of TP63 and SCL7A11 proteins in breast tissue samples from the tumor group and the normal group. First, the collected tissue samples were fixed in 10% neutral-buffered formalin and embedded in paraffin. Subsequently, 4 μm thick sections were cut from the paraffin-embedded blocks and mounted on glass slides. Antigen retrieval was performed to enhance antigenicity, followed by blocking to minimize nonspecific binding. The sections were then incubated overnight at 4°C or for a specified duration with primary antibodies targeting TP63 (diluted 1:50) and SCL7A11 (diluted 1:100). Afterward, the sections were washed and incubated with secondary antibodies conjugated to a reporter enzyme. Visualization was achieved using a chromogenic substrate, resulting in a visible precipitate at the sites of antigen-antibody interaction. Counterstaining with hematoxylin was performed to improve tissue visibility. Subsequently, stained sections were examined under an optical microscope to assess TP63 and SCL7A11 expression levels. Staining intensities were compared between the tumor group and the normal group to determine any differential expression patterns. Additionally, ImageJ software was used to analyze the positive area. Identification of tissue subtypes in BRCA patients based on the single-cell RNA-Seq dataset GSE76078.

### Data sources

The TCGA breast cancer datasets were downloaded using the UCSC Xena browser and included 1,091 tumor tissue samples and 113 normal tissue samples. The breast cancer typing data was downloaded with the R package TCGAbiolinks, and the GSE96058 and GSE25066 datasets were downloaded from NCBI.

### Screening and identification of DEGs

Differential analysis of genes obtained from the training datasets was performed with the limma package. The criteria for selecting DEGs were |logFoldChange| ≥ 1 and *P*-value < 0.05. Ferroptosis-related signature genes and DEGs reported in the previous literature and the FerrDb website were searched to determine candidate ferroptosis-related factors (http://www.zhounan.org/ferrdb/) (7).

### Construction and verification of a prognostic risk model based on ferroptosis-related signature genes

The survival package (R 4.0) was used to perform survival analysis on candidate ferroptosis-related factors (*P*-value < 0.05). A PPI (Protein-protein interaction) network was constructed for the ferroptosis-related factors with the String database. In correlation analysis was using the Hmisc package (R 4.0).

Lasso dimensionality reduction was performed with the glmnet package. The number of iterations was 1000, and cross-validation was performed to construct a scoring model of the risk for breast cancer ferroptosis. The risk score for each patient was calculated according to the risk formula. Risk score = gene A expression × β1 + gene B expression × β2 +……gene expression × βn (βn represents the coefficient of the corresponding gene). ROC analysis and survival analysis were performed using the pROC package and survival package (R 4.0). Additionally, other clinical factors were analyzed by univariate and multivariate COX regression analysis. LASSO Cox regression analysis by glmnet package (R 4.0). The clinical data of the training set included age, menopausal status, cancer stages, T stages, lymph node metastasis, and PAM50 classification. The clinical data of the validation set included age, lymph node metastasis, and PAM50 classification.

### Functional and pathways enrichment analysis of the identified DEGs

The limma package (R 4.0) was used to perform differential analysis of the low- and high-risk groups of the training set and the validation set, and the clusterProfiler package (R 4.0) was used to perform functional and pathways enrichment analysis of the DEGs. And take the intersection of the enrichment analysis results of the training set and the validation set. The cutoff *P*-value for the KEGG pathways enrichment and GO terms enrichment analysis were 0.05.

### ssGSEA analysis

Single sample gene set enrichment analysis (ssGSEA) is an algorithm used to assess the degree of tumor immune infiltration. The datasets were evaluated using ssGSEA with the GSVA package and GSEABase package (R 4.0). Genetic information about the immune cells was obtained from the literature (7).

### Analysis of differences in mutations in ferroptosis-related factors between risk groups and analysis of treatment differences

The maftools package and the GenVisR package were used to divide the validation set data into risk groups by risk score. Mutation information was from the mutation data. The screening condition was mainRecurCutoff = 0.05.

The immunotherapy dataset was used to obtain data about immunotherapy (14). TCGA dataset grouping constructs low- and high-risk group data. The difference in immunotherapy between risk groups was predicted with the submap model of the Genepattern website.

Using the GSE25066 dataset, the ggplot2 and Survival packages were used to analyze differences in the prediction of chemotherapy sensitivity between risk groups, and the Wilcoxon test was used to analyze differences in the expression of genes identified in the drug resistance samples.

### Drug sensitivity analysis

We conducted a comprehensive drug resistance assessment of genes using data from the GDSC drug database. We integrated mRNA expression data with drug sensitivity data, followed by performing Pearson correlation analysis to assess the relationship between the mRNA expression levels of selected ferroptosis-related genes and the corresponding IC50 values of drugs. To enhance statistical rigor, we adjusted the p-values using the False Discovery Rate (FDR) correction method.

## Results

### DEG screening

**Figure 1:**
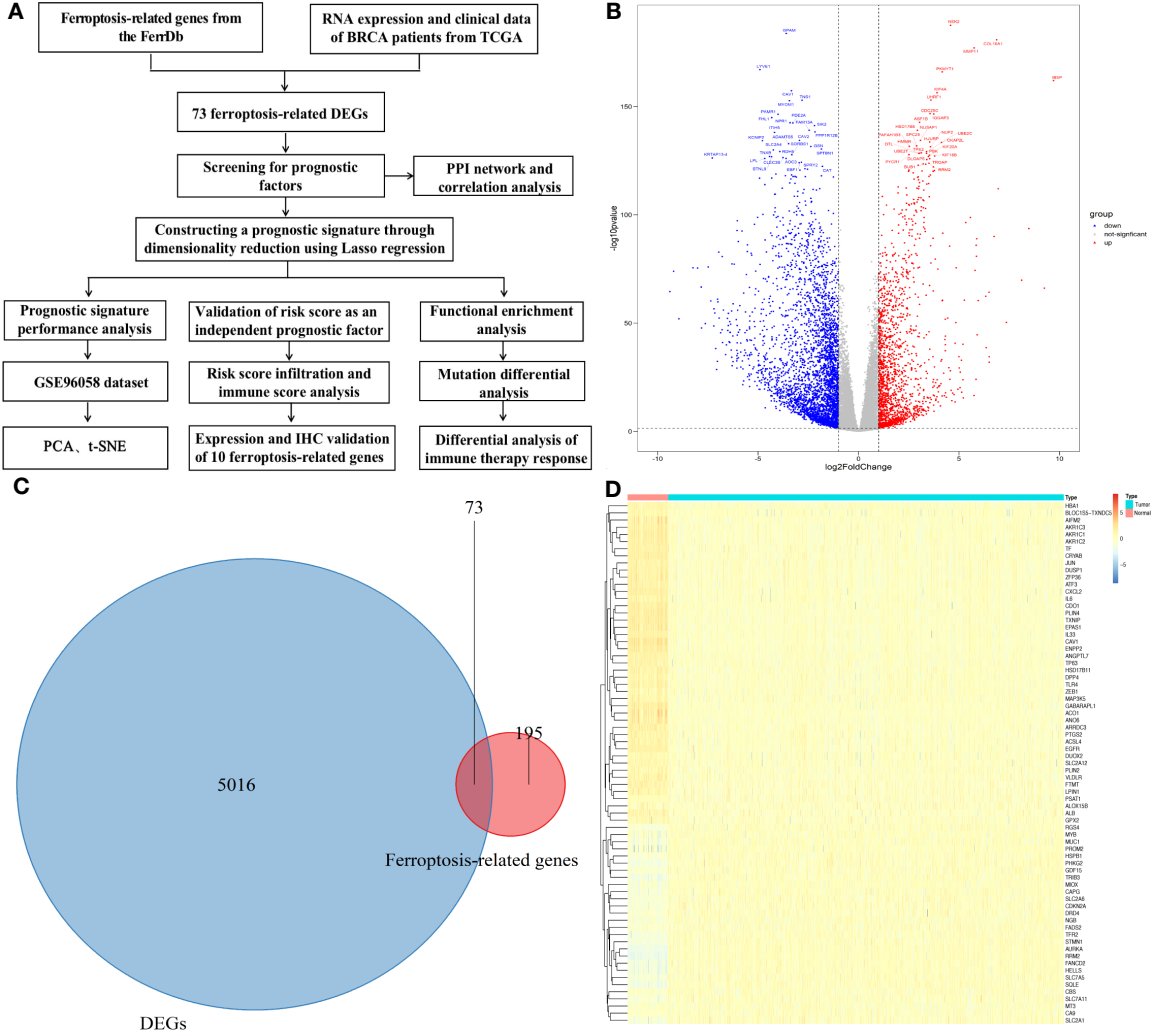
Differential genes (DGEs) analysis. **(A)**The study flowchart. **(B)** Volcano plot of the differentially expressed genes identified from the TCGA-BRCA database. **(C)** Venn diagram of the differentially expressed genes and ferroptosis-related genes. **(D)** Heatmap showing expression of the 73 overlapping genes between tumor and normal samples.

**Figure 1A** is the flowchart of our study. The difference in genes expression between breast cancer tumor and adjacent tissue samples in the TCGA datasets were analyzed, and 5089 DEGs were obtained: 3347 genes were downregulated and 1742 genes were upregulated (**Figure 1B** and **Tables S2, S3**). Seventy-three candidate ferroptosis factors were common between the screened ferroptosis-related signature genes and DEGs, and heatmaps were used to visualize them (**Figures 1C**, **D**).

### Identifying ferroptosis-related factors and constructing a prognostic model for breast cancer

**Figure 2:**
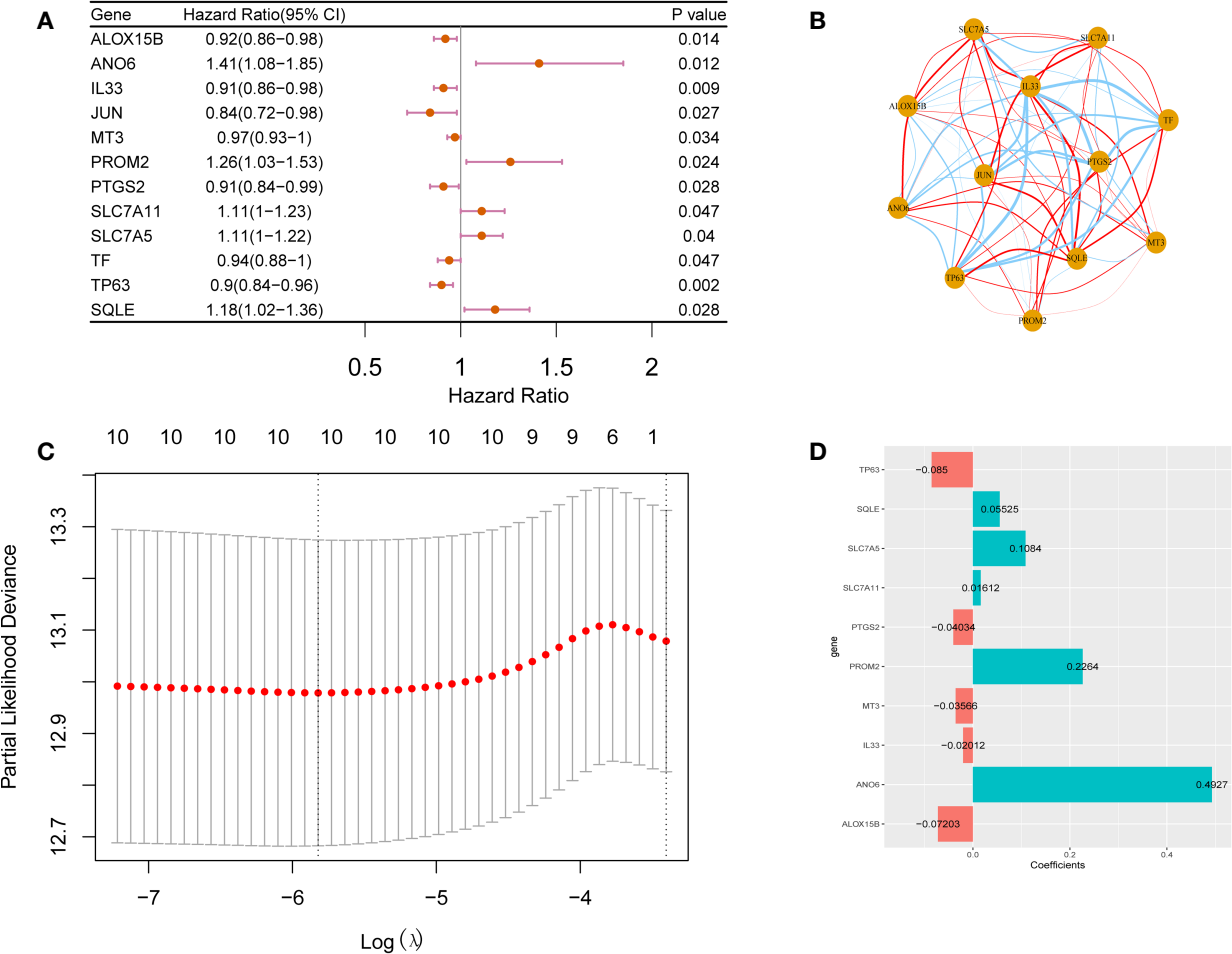
Prognostic factors analysis for breast cancer. Forest plot **(A)** and correlation analysis network plot **(B)** of 12 prognostic factors for breast cancer. Prognostic factors associated with breast cancer are CV chart **(C)** and risk coefficient bar chart **(D)**.

Univariate Cox regression analysis of the 73 candidate ferroptosis-related factors revealed 12 potential prognostic factors related to breast cancer (**Table S4**). These 12 genes are closely related to the prognosis of breast cancer patients. Among them, *ANO6*, *PROM2*, *SLC7A11*, *SLC7A5* and *SQLE* are risk genes (HR > 1.0, *p <*0.05) and *ALOX15B*, *IL33*, *JUN*, *MT3*, *PTGS2*, *TF* and *TP63* are protective genes (HR < 1.0, *p <*0.05, **Figure 2A**). **Figure 2B** shows the correlation between the 12 prognostic ferroptosis-related signature genes (ANO6, PROM2, SLC7A11, SLC7A5, SQLE, ALOX15B, IL33, JUN, MT3, PTGS2, TF, and TP63).

To construct a prognostic model comprising ferroptosis-related factors for breast cancer, and LASSO Cox regression analysis on the 12 identified breast cancer prognosis-related factors. From these 12 factors, 10 factors (i.e., *TP63*, *SQLE*, *SLC7A5*, *SLC7A11*, *PTGS2*, *PROM2*, *MT3*, *IL33*, *ANO6*, *ALOX15B*) that contributed the most to the prognosis of breast cancer patients were screened out based on an optimal value of λ (**Figures 2C**, **D**). The following formula was used to construct a breast cancer prognostic model comprising ferroptosis-related factors:

Riskscore=TP63*-0.085+SQLE*0.05525+SLC7A5*0.1084+SLC7A11*0.01612+PTGS2*-0.04034+PROM2*0.2264+MT3*-0.03566+IL33*-0.02012+ANO6*0.4927+ALOX15B*-0.07203.

The median risk score was used as the cutoff value, patients in the TCGA datasets were divided into the low- and the high-risk group (**Figure S1A**). Survival analysis suggested that the low-risk group had a longer survival time (**Figure S1B**, *p* < 0.01).

### Evaluation of 10 ferroptosis-related signatures genes as independent prognostic factors in breast cancer patients

**Figure 3:**
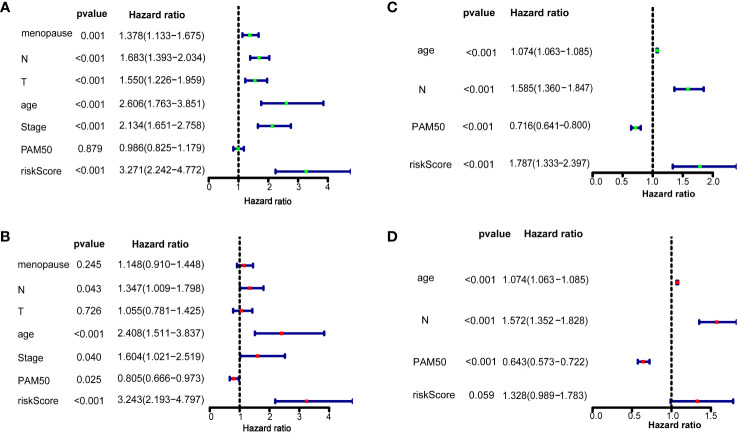
Risk score as an independent prognostic factor for breast cancer Results of overall survival in the training dataset and validation dataset using univariate **(A, B)** and multivariate Cox regression analyses **(C, D)**.

We further confirmed whether the 10 ferroptosis-related gene signatures were independent prognostic factors by univariate and multivariate COX regression analysis. In the training dataset, univariate COX regression analysis suggested that M (HR=1.378, 95%CI = 1.133-1.675, *p*=0.001), N (HR=1.683, 95%CI = 1.393-2.034, *p* < 0.001), T (HR=1.550, 95%CI = 1.226-1.959, *p* < 0.001), age (HR=2.606, 95%CI = 1.763-3.851, *p* < 0.001), stage (HR=2.134, 95%CI = 1.651-2.758, *p* < 0.001), and risk score (HR = 3.271, 95% CI = 2.242–4.772, *p* < 0.001; **Figure 3A**) and the multivariate Cox regression suggested that age (HR=1.074, 95%CI = 1.063-1.085, *p* < 0.001), N (HR=1.585, 95%CI = 1.360-1.847, *p* < 0.001), PAM50 (HR=0.736, 95%CI = 0.641-0.800, *p* < 0.001), and risk score (HR = 1.787, 95% CI = 1.333–2.397, *p* < 0.001, **Figure 3C**). In the validation set, univariate COX regression analysis showed that N (HR=1.347, 95%CI = 1.009-1.798, *p* = 0.043), age (HR=2.408, 95%CI = 1.511-3.837, *p* < 0.001), stage (HR=1.604, 95%CI = 1.021-2.519, *p* = 0.040), PAM50 (HR=0.805, 95%CI = 0.666-0.973, *p* = 0.025) and risk score (HR = 3.243, 95% CI = 2.193–4.797, *p* < 0.001; **Figure 3B**) and the multivariate Cox regression suggested that age (HR=1.074, 95%CI = 1.063-1.085, *p* < 0.001), N (HR=1.572, 95%CI = 1.352-1.828, *p* < 0.001), PAM50 (HR=0.643, 95%CI = 0.573-0.722, *p* < 0.001), and risk score (HR = 1.328, 95% CI = 0.989–1.783, *p* = 0.059, **Figure 3D**). All these results suggested that these 10 ferroptosis-related gene signatures are significantly related to the prognosis of breast cancer patients.

### Functional enrichment analysis of DEGs in the risk groups and assessment of immune function in the risk groups

To clarify the enrichment pathways and biological functions of the identified prognostic ferroptosis-related signature genes, functional enrichment analysis was performed on the DEGs between the risk groups in the training set and validation set (**Tables S5, S6**). A total of 620 DEGs were identified in the training dataset (**Table S7**) and 817 DEGs were identified in the validation dataset (**Table S8**). The training set DEGs were enriched in 150 GO terms (**Figure S2A** and **Table S9**) and 8 KEGG pathways (**Figure S2B** and **Table S10**), the KEGG pathways were enriched in cytokine-cytokine receptor interaction (hsa04060), viral protein interaction with cytokine and cytokine receptor (hsa04061), neuroactive ligand-receptor interaction (hsa04080), staphylococcus aureus infection (hsa05150), tyrosine metabolism (hsa00350), PPAR signaling pathway (hsa03320), arachidonic acid metabolism (hsa00590) and IL-17 signaling pathway (hsa04657). And the validation set DEGs were enriched in 650 GO terms (**Figure S2C** and **Table S11**) and 18 KEGG pathways (**Figure S2D** and **Table S12**), the KEGG pathways were enriched in Cytokine-cytokine receptor interaction (hsa04060), viral protein interaction with cytokine and cytokine receptor (hsa04061), hematopoietic cell lineage (hsa04640), ECM-receptor interaction (hsa04512), PPAR signaling pathway (hsa03320), primary immunodeficiency (hsa05340), focal adhesion (hsa04510), chemokine signaling pathway (hsa04062), renin secretion (hsa04924), etc. Between the two sets, there were 85 overlapping GO terms (**Table S13**) and 4 overlapping KEGG pathways (**Table S14**). The main functions and pathways that were enriched in the PPAR signaling pathway, cytokine receptor function, tyrosine metabolism, response to chemokines, cell adhesion molecules, extracellular matrix structural components, glycosaminoglycan binding, and heparin-binding.

**Figure 4:**
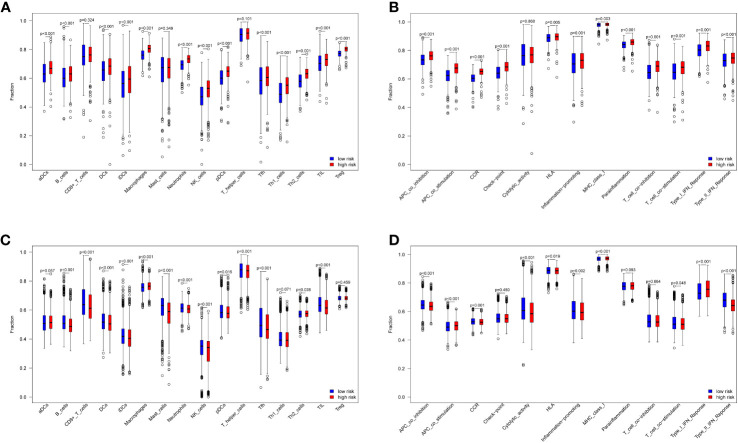
Immune cell and immune-related functions between low- and high-risk groups. 16 immune cells and 13 immune-related functions in the TCGA datasets **(A, B)** and GEO datasets **(C, D)**.

The ssGSEA algorithm was used to compare immune cell infiltration in the risk groups. The combined results from the TCGA and GEO datasets showed that the high-risk group had significant higher B cell, DCs, iDCs, macrophage, neutrophil, pDC, NK cell, Tfh cell, Th2 cell, and TIL cell infiltration than the low-risk group (*p* < 0.05, **Figures 4A, C**). Immune cell infiltration was generally higher in the high-risk group. In addition, immune-related functions, including APC co-stimulation, APC co-inhibition, HLA, CCR, promotion of inflammation, type I IFN response, type II IFN response, MHC class I and T cell co-stimulation were significantly upregulated in the high-risk groups (*p* < 0.05, **Figures 4B, D**). Overall, these 10 ferroptosis-related genes were also found to be related to the immune status of breast cancer.

### Differences in mutations in ferroptosis-related factors between the low- and high-risk groups and differences in treatment sensitivity

*MUC4* and *NEB* mutations were detected in the low-risk group, but not in the high-risk group, of the training set. In the high-risk group of the training set, *FLG*, *NCOR1*, *SPTA1*, *DMD*, *PTEN*, *ZFHX4*, *CSMD3*, *MUC17*, and *FAT3* mutations were detected (**Figure S3A**), but these were not detected in the low-risk group (**Figure S3B**). *PIK3CA*, *TP53*, *CDH1*, *TTN*, *MUC16*, *GATA3*, *MAP3K1*, *KMT2C*, *HMCN1*, *DSH2A*, *RYR2*, *SYNE1* mutations were detected in the low- and high-risk groups. Additionally, there was a difference in the proportion of *CDH1* between the low- and high-risk groups. Our prediction analysis of immunotherapy outcome showed that the anti-PD1 (programmed cell death protein 1) treatment may be less effective in the high-risk group (**Table S15**).

**Figure 5:**
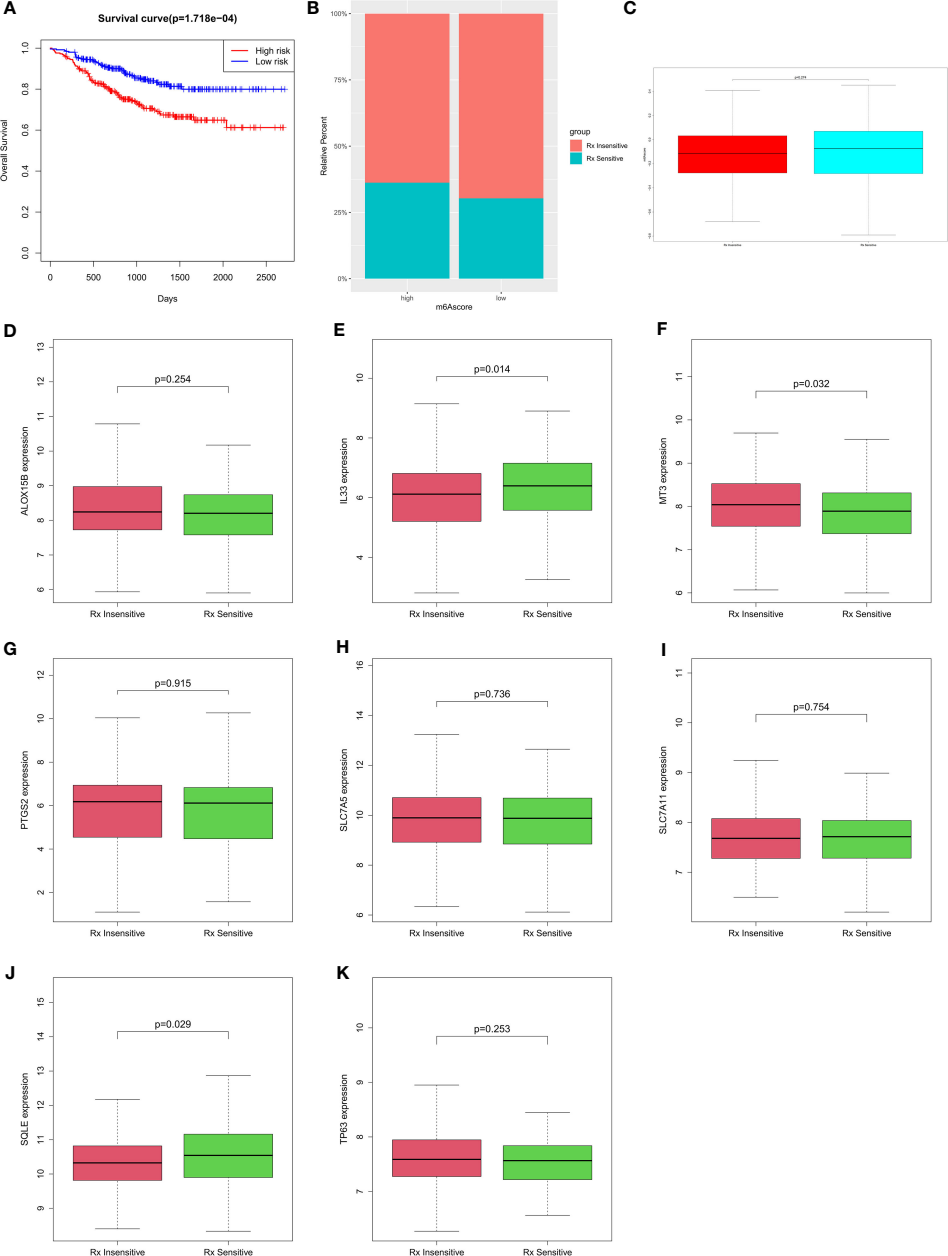
Differences in the prediction of chemotherapy sensitivity according to risk **(A)** Prognostic survival analysis of high and low risk groups; **(B)** Ratio of treatment insensitives and treatment sensitives in the risk group of the high- and low-risk group; **(C)** Differences in riskscore between Treatment Sensitive and Treatment Insensitive; **(D-K)** Differences in gene sensitivity therapy among different genes.

There was a significant difference in overall survival between the low- and high-risk groups (**Figure 5A**), and this was reflected by the prognostic model. In the high-risk group, 36.22% of the samples were Treatment sensitive and 63.78% were Treatment insensitive, and in the low-risk group, 30.31% were Treatment sensitive and 69.69% were Treatment insensitive (**Figure 5B** and **Table S16**). There was an insignificant difference in risk score between the Treatment-sensitive and Treatment-insensitive samples (**Figure 5C**). Then, we analyzed the genes expression in the prognostic model according to drug resistance. The results showed that the *IL33*, *MT3*, and *SQLE* genes had significantly different expressions in the Treatment-sensitive and Treatment-insensitive samples, and *ALOX15B*, *PTGS2*, *SLC7A5*, *SLC7A11*, and *TP63* expression between Treatment-sensitive and Treatment-insensitive samples were not statistically significantly different. (**Figures 5D-K** and **Table S17**).

Finally, we analyzed the drug resistance of genes based on the GDSC drug database (https://www.cancerrxgene.org/faq). The results showed that *PROM2*, *TP63*, *PTGS2*, *MT3*, *ANO6*, *SQLE*, and *SLC7A11* were associated with drug response. *PROM2* and *TP63* were sensitive to gefitinib, afatinib, lapatinib, erlotinib, cetuximab, and docetaxel. Notably, the expression of *SLC7A11* was positively correlated with Shikonin, QL-XII-61, QL-X-138 (**Figure S4**).

### Validation of ferroptosis-related genes expression

**Figure 6:**
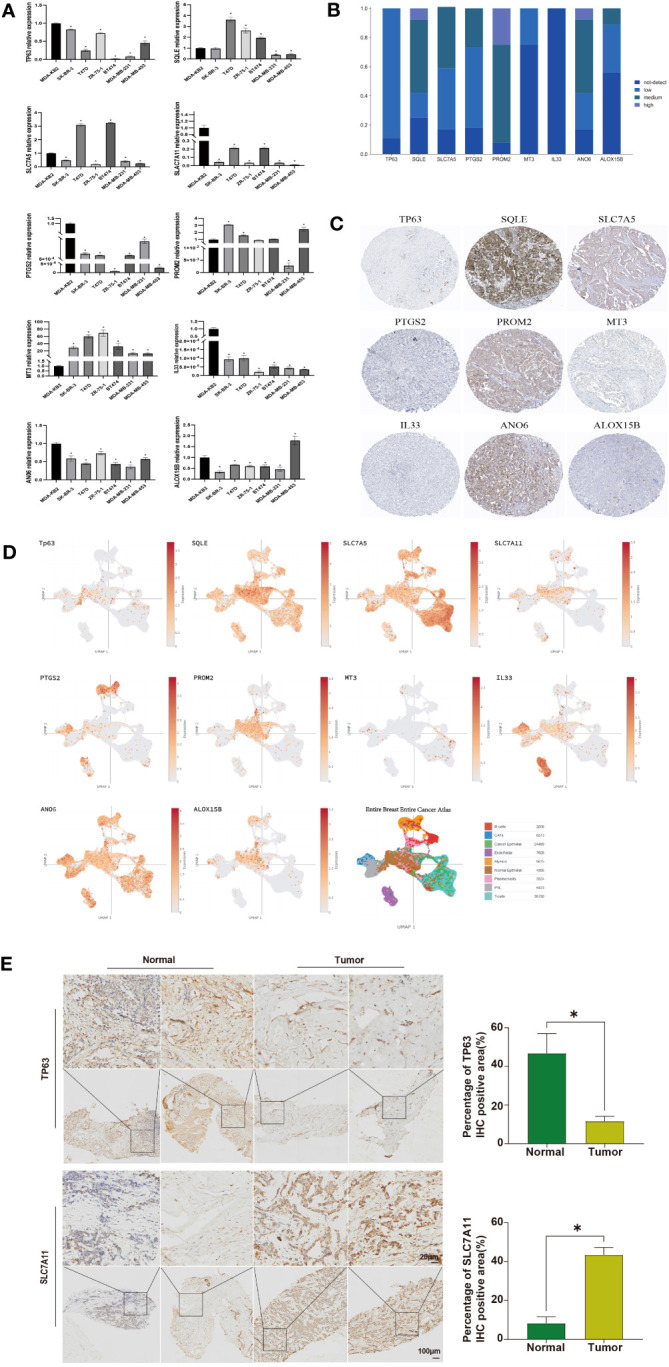
Analysis of Expression in Cell Lines and IHC Analysis. **(A)** RT-qPCR detection of the expression of ferroptosis-related genes in breast cancer cell lines, with β-actin as a control; **(B)** Collection of IHC staining information for ferroptosis-related genes; **(C)** Representative IHC staining for ferroptosis-related genes; **(D)** The expression level of ferroptosis-related genes. Identification of tissue subtypes in BRCA patients based on the single-cell RNA-Seq dataset GSE76078; **(E)** IHC analysis of TP63 and SCL7A11 protein expression in human breast tissue samples. Statistically significant differences are represented by ‘*’, *P*<0.05.

Finally, we validated the expression levels of these ten ferroptosis-related genes in human breast cancer cell lines. Compared to the normal breast cell line MDA-KB2, TP63, SLC7A11, PTGS2, IL33, and ANO6 were downregulated in other cell lines. SQLE showed high expression in T47D, ZR-75-1, and BT474, while it was lowly expressed in MDA-MB-231 and MDA-MB-453 (**Figure 6A**). SLC7A5 exhibited low expression in SK-BR-3, ZR-75-1, MDA-MB-231, and MDA-MB-453, but high expression in T47D and BT474. PROM2 demonstrated high expression in SK-BR-3, T47D, and MDA-MB-453. ALOX15B displayed low expression in SK-BR-3, T47D, ZR-75-1, BT474, and MDA-MB-231, but high expression in MDA-MB-453 (**Figure 6A**). To further validate our findings, we utilized IHC analysis. IHC data were retrieved from the HPA database to examine the protein levels associated with the signature ARG. It was observed that 8/9, 9/12, 10/12, 9/11, 11/12, 3/12, 10/12, and 4/9 BRCA samples expressed TP63, SQLE, SLC7A5, PTGS2, PROM2, MT3, ANO6, and ALOX15B, respectively (**Figure 6B**). Notably, through IHC analysis, it was observed that these eight ferroptosis-related proteins were localized in the cytoplasm and/or membrane of the BRCA samples (**Figure 6C**). However, IL33 was not detected in any of the BRCA samples (**Figures 6B, C**). Due to the lack of IHC staining for SLC7A11 in the BRCA samples, the protein expression was not estimated. In addition, we also focused on the expression of these 10 ferroptosis-related genes in breast cancer samples in single cell data analysis. The expression patterns of SQLE, SLC7A5, and ANO6 are similar in different cells (**Figure 6D**). To validate the protein-level expression of the aforementioned genes, we selected two genes that exhibited significant differences in expression levels. Immunohistochemical staining was performed on human breast tissue samples obtained from the tumor group and the normal group. The results of the immunohistochemical staining revealed distinct staining patterns for TP63 and SCL7A11 in different groups. Statistical analysis further demonstrated a significant decrease in TP63 expression in the tumor group compared to the normal group. Conversely, the expression of SCL7A11 was significantly increased in the tumor group when compared to the normal group (**Figure 6E**, *P*<0.05).

## Discussion

In our study, we systematically studied the expression of 73 ferroptosis-related genes in breast cancer and their relationship with overall survival. And based on the data of the TCGA dataset, a novel model containing 10 ferroptosis-related genes was constructed to predict the prognosis of breast cancer, and it was validated in the GEO dataset. As well as in the functional enrichment analysis results suggested that immune-related functions were enriched. Overall, the findings suggest that according to the model, breast cancer patients classified as high-risk groups lived less than low-risk groups.

In the present study, 10 significant ferroptosis-related factors associated with breast cancer were used to construct the model for prediction of prognosis: *Tp63*, *SQLE*, *SLC7A5*, *SLC7A11*, *PTGS2*, *PROM2*, *MT3*, *IL33*, *ANO6*, and *ALOX15B*. According to FerrDb (http://www.zhounan.org/ferrdb/) (15), TP63 and SLC7A11 are inhibitors of ferroptosis, while *SQLE*, *ANO6*, *IL33*, and *ALOX15B* may promote ferroptosis. So far, *SLC7A5* and *MT3* have not been reported to be associated with ferroptosis.

*SLC7A11* is closely related to resistance to ferroptosis inducers. *SLC7A11* is highly expressed in tumour samples and the ectopic expression of *SLC7A11* inhibits by p533KR-induced ferroptosis in human cancer cells (16, 17). *SLC7A11* directly interacts with *ALOX12*, resulting in the inhibition of *ALOX12* activity. A recent report suggested that *ALOX12* is essential for p53-mediated ferroptosis (18). There are six *ALOX* (arachidonic acid lipoxygenase) genes in humans: *ALOXE3*, *ALOX12*, *ALOX12B*, *ALOX15*, *ALOX15B*, and *ALOX5*. Silencing the *ALOX* gene makes cells resistant to ferroptosis. By silencing *ALOX15B* and *ALOXE3*, erastin-induced cell death can be rescued, which supports the hypothesis that ferroptosis requires lipoxygenase (19). *TP63* is a member of the *Tp53* family (20). *TP63* via its byproducts ΔNp63 regulates the self-renewal of progenitor cells in epithelial tissue, which has a dominant-negative effect on other isoforms of the *Tp53* family and exerts tumorigenic functions (21). *TP63* amplification up-regulates glutathione metabolism and promotes tumorigenesis. ΔNp63α inhibits oxidative stress by regulating glutathione metabolism. ΔNp63α is an important cell guard against oxidative stress (including ferroptosis) (22). The expression of *TP63* was positively correlated with the expression levels of glutathione metabolism-related genes, including *SLC7A11*, *GCLC*, and *GSS* (23). *ANO6* is activated during the ferroptosis process induced by erastin and *RSL3*. The activation of *ANO6* is a key component of the ferroptosis cell death process. Inhibition prevents the ferroptosis cell death induced by RSL3/erastin (24). *PROM2* is induced by ferroportin, and Prominin2 promotes the ferroptosis resistance of breast cancer cells. In terms of mechanism, prominin2 promotes the formation of ferritin-containing exosomes and multivesicular bodies (MVB), transports iron out of cells, and inhibits ferroptosis (25).

Studies have shown that *PTGS2* may promote ferroptosis, and the up-regulation of *PTGS2* is a suitable marker for lipid peroxidation that occurs during ferroptosis regulated by *GPX4*. GPX4 is an established central regulator of ferroptosis and can induce ferroptosis in mouse tumor xenografts (26). Ferrostatin-1 (Fer-1) is an inhibitor of ferroptosis, regarding the immunogenicity of ferroptosis. Fer-1 prevents the up-regulation of *IL-33*, which is an alarm related to necroptosis and prevents macrophage infiltration and Klotho down-regulation (27). Therefore, the role of *SLC7A5* and *MT3* genes in ferroptosis can be explored in the future.

In recent years, although the relationship between the occurrence and development of tumors and ferroptosis has been intensively studied, the mechanism by which genes regulate breast tumor occurrence and development by affecting ferroptosis remains unclear.

In this study, we evaluated the expression levels of 10 ferroptosis-related genes in breast cancer cells and further validated their protein expression using IHC analysis. Our findings revealed distinct expression patterns among these ferroptosis-related genes in breast cancer cells. The IHC analysis provided further validation of the protein expression of these genes in breast cancer tissue samples. Furthermore, we performed single-cell data analysis to visualize the expression levels of these genes in different cells, with the aim of identifying patterns associated with breast cancer prognosis. These genes may potentially be involved in the pathogenesis of breast cancer or interact with other genes, but further research is needed to confirm their functions and biological significance. Although we did not observe clear differential expression patterns, our study provides a starting point for further exploration of the roles of these 10 genes in breast cancer. Deeper investigation into the functions and regulatory mechanisms of these genes, as well as their association with breast cancer prognosis, will contribute to a better understanding of the pathological processes in breast cancer and provide new clues for treatment and prognosis assessment. These results indicate a potential clinical relevance of these genes in breast cancer progression and treatment response.

It is worth noting that the dysregulation of ferroptosis-related genes may contribute to the development of chemoresistance in breast cancer. Previous studies have demonstrated the involvement of ferroptosis in cancer biology and its therapeutic implications (28–30). In conclusion, our study provides a comprehensive analysis of the expression patterns of 10 ferroptosis-related genes in breast cancer cells, supported by IHC validation in tumor tissues. These findings contribute to the growing understanding of ferroptosis dysregulation in breast cancer and its potential implications in clinical management. Further research is necessary to fully unravel the functional roles of these genes and their therapeutic significance in breast cancer.

In the present study, GO terms and KEGG pathway analysis of the DEGs identified between the training set and the validation set shows that extracellular matrix decomposition, humoral immune response, cytokine receptor function, chemokine receptor binding, and PPAR signaling pathways, among other pathways, were enriched. Accordingly, studies have shown that the shedding of extracellular mechanisms leads to the accumulation of oxidative stress and ferroptosis (31). Further, the PPAR signaling pathway plays a central role in lipid metabolism (32), and iron-dependent accumulation of lipid hydroperoxides leading to a lethal level is characteristic of ferroptosis (33). It is also known that the dysregulation of extracellular matrix deposition promotes breast cancer aggressiveness by maintaining important growth, invasion, and survival-related signaling pathways (34, 35). Glycosaminoglycans are biomarkers and targets for the diagnosis, prognosis, and treatment of breast cancer and play a key role in their growth (36, 37). Therefore, these functional enrichment analysis results suggest that ferroptosis-related genes may promote breast cancer progression by regulating some signaling pathways and the immune system at the early stage of breast cancer.

The potential association between cancer immunity and ferroptosis remains to be further elucidated. In our research results, we found that compared with the high- and the low-risk group had significantly lower B cell, DC, iDC, macrophage, neutrophil, NK cell, pDC, Tfh, Th2 cell, and TIL cell infiltration. Tumor-infiltrating B cells (TIB) have been shown to promote tumor cell lysis and apoptosis (38). Neutrophil infiltration is associated with increased overall survival in breast cancer and is an independent prognostic factor (39). Additionally, dendritic cells (DCs) are known to have a role in initiating immune mechanisms. Studies suggest that defective DCs function in patients with early-stage breast cancer is one of the important factors in tumor progression (40). Consistent with our findings, in our ferroptosis-related prognostic model, there was a significant difference in the proportion of DCS in breast cancer patients among the risk groups. In conclusion, dysfunction of the immune response in the peri-tumor environment may be one of the reasons for the low-risk score.

Through univariate and multivariate Cox regression analysis, it was found that the model constructed in this study can predict prognosis well, and reliable results were obtained in constructing a risk score model. At the same time, we should acknowledge some limitations of this study. All results are based on public datasets and prospective clinical validation should be required in the future. In conclusion, 10 ferroptosis-related gene signatures were identified as independent prognostic significance in breast cancer. It also provides potential biomarkers and models for future personalized medicine and immune-related work in the treatment of breast cancer patients.

## Conclusion

In summary, our findings highlight the crucial role of ferroptosis-related genes in breast cancer prognosis. By developing a novel prognostic risk prediction model that combines these genes and immune cell infiltration information, we offer a promising approach for assessing the prognostic value of ferroptosis-related genes in breast cancer. Additionally, our validation study confirmed the expression levels of ferroptosis-related genes in human breast cancer cell lines and assessed the expression of ferroptosis-related proteins in cancer specimens using IHC analysis. Further studies are warranted to validate and explore the clinical implications of this model, potentially improving treatment strategies and patient outcomes.

## Data availability statement

The original contributions presented in the study are included in the article/**Supplementary Material**, further inquiries can be directed to the corresponding authors.

## Ethics statement

The studies involving humans were approved by Ethics Committee of the First Affiliated Hospital of the Army Medical University. The studies were conducted in accordance with the local legislation and institutional requirements. The participants provided their written informed consent to participate in this study.

## Author contributions

LZ: Data curation, Formal analysis, Writing – original draft, Writing – review & editing. TZ: Data curation, Formal analysis, Writing – original draft, Writing – review & editing. XW: Data curation, Formal analysis, Writing – original draft, Writing – review & editing. HT: Formal analysis, Software, Writing – original draft. PG: Formal analysis, Writing – review & editing. QC: Writing – review & editing. CC: Writing – review & editing. YZ: Writing – review & editing. SW: Formal analysis, Project administration, Visualization, Writing – original draft, Writing – review & editing. XQ: Formal analysis, Funding acquisition, Project administration, Visualization, Writing – original draft, Writing – review & editing. NS: Formal analysis, Project administration, Visualization, Writing – original draft, Writing – review & editing.

## Supplementary material

Supplementary Figure 1 Prognostic analysis of the 12-gene model in the TCGA datasets **(A)** The distribution of risk score in the TCGA datasets. **(B)** Kaplan-Meier survival analysis of OS between the risk group in the TCGA datasets.

Supplementary Figure 2 Functional enrichment of DEGs identified between the risk groups. GO terms enrichment analysis in the TCGA datasets **(A)** and in the GEO datasets **(C)**. KEGG pathways enrichment analysis in the TCGA datasets **(B)** and in the GEO datasets **(D)**.

Supplementary Figure 3 Analysis of differences in mutations in ferroptosis factors according to risk **(A)** Low-risk group; **(B)** High-risk group.

Supplementary Figure 4 Drug resistance analysis based on GDSC drug data.
