# Supplementary material for: Construction of a ferroptosis-based prognostic model for breast cancer helps to discriminate high/low risk groups and treatment priority
Source: Front Immunol. 2023 Dec 13;14:1264206. doi: 10.3389/fimmu.2023.1264206 (PMC10751362; doi:10.3389/fimmu.2023.1264206)
Supplement: Supplementary Figure 1 — Prognostic analysis of the 12-gene model in the TCGA datasets (A) The distribution of risk score in the TCGA datasets. (B) Kaplan-Meier survival analysis of OS between the risk group in the TCGA datasets. [file DataSheet_1.zip › Supplementary-/SupplementaryTable 1.docx]

|  | Patients | Number | Group | Age | Molecular Subtype | Histological Type | Ki67 |
| --- | --- | --- | --- | --- | --- | --- | --- |
| TP63 | 1 | 3608902 | Normal | 26 | - | FB | - |
|  | 2 | 8135127 | Normal | 31 | - | Cyclomastopathy | - |
|  | 3 | 3629657 | Tumor | 35 | LuminalB | NSIBC | 30%+ |
|  | 4 | 3620284 | Tumor | 56 | Her2+ | IBC | 15%+ |
| SLC7A11 | 5 | 1681890 | Normal | 48 | - | FB | - |
|  | 2 | 8135127 | Normal | 31 | - | Cyclomastopathy | - |
|  | 6 | 3873272 | Tumor | 58 | LuminalB | IBC | 20%+ |
|  | 7 | 3775587 | Tumor | 49 | TNBC | NSIBC | 60%+ |

Table S1 Clinicopathological Characteristics of Patients.

Abbreviations:TNBC, triple negative breast cancer; FB, fibroadenoma of breast; IBC, invasive breast carcinoma; NSIBC, nonspecific invasive breast carcinoma.
