# Supplementary figures and images for: Construction of a ferroptosis-based prognostic model for breast cancer helps to discriminate high/low risk groups and treatment priority
Source: Front Immunol. 2023 Dec 13;14:1264206. doi: 10.3389/fimmu.2023.1264206 (PMC10751362; doi:10.3389/fimmu.2023.1264206)

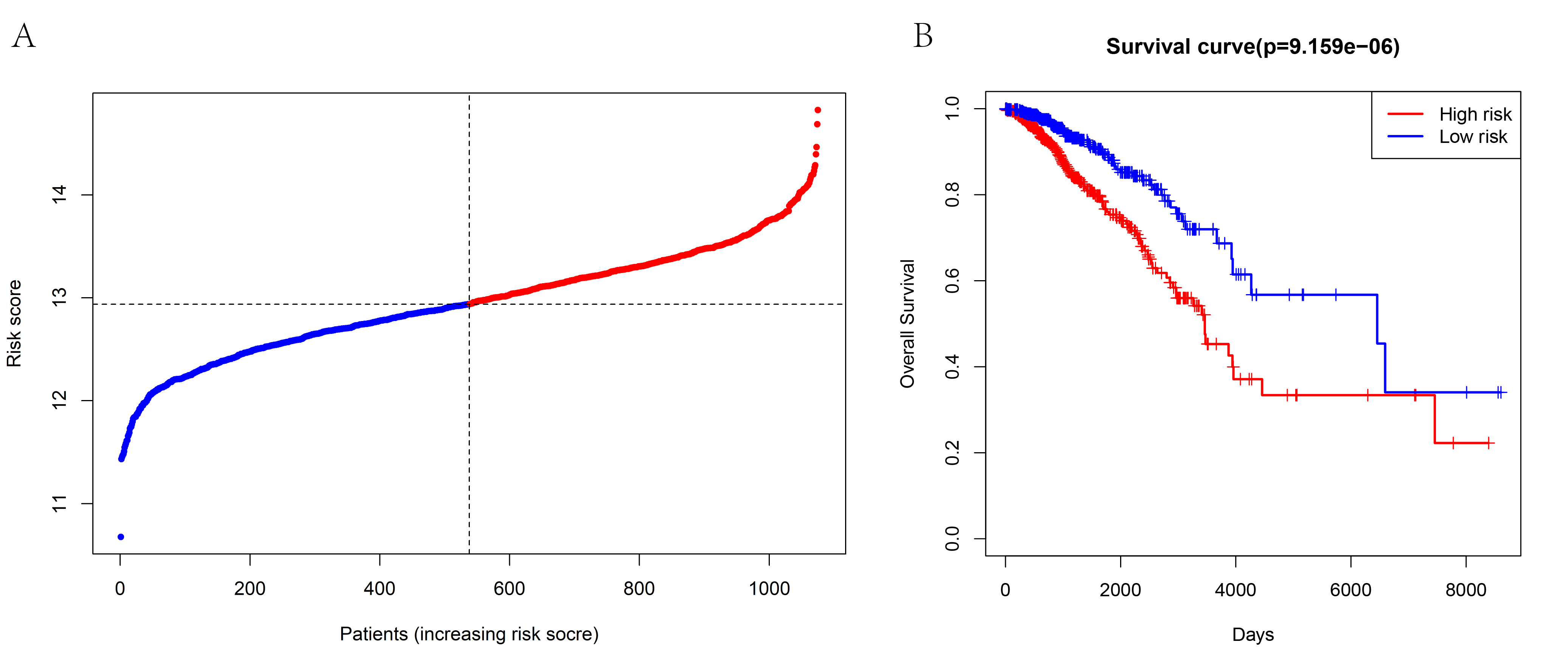

Supplement: Supplementary Figure 1 — Prognostic analysis of the 12-gene model in the TCGA datasets (A) The distribution of risk score in the TCGA datasets. (B) Kaplan-Meier survival analysis of OS between the risk group in the TCGA datasets. [file DataSheet_1.zip › Supplementary-/Supplementary Figure 1.tif]

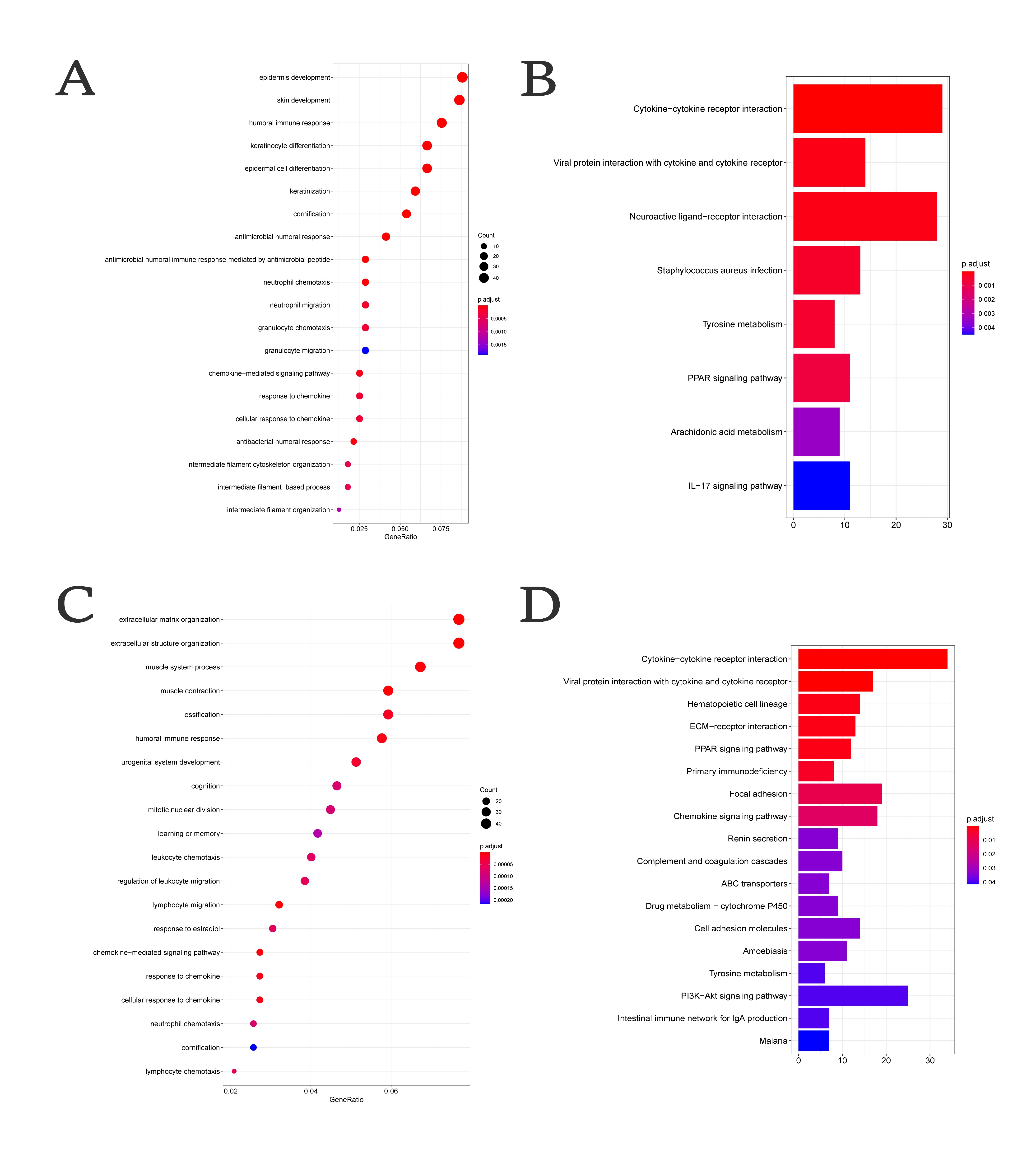

Supplement: Supplementary Figure 1 — Prognostic analysis of the 12-gene model in the TCGA datasets (A) The distribution of risk score in the TCGA datasets. (B) Kaplan-Meier survival analysis of OS between the risk group in the TCGA datasets. [file DataSheet_1.zip › Supplementary-/Supplementary Figure 2.tif]

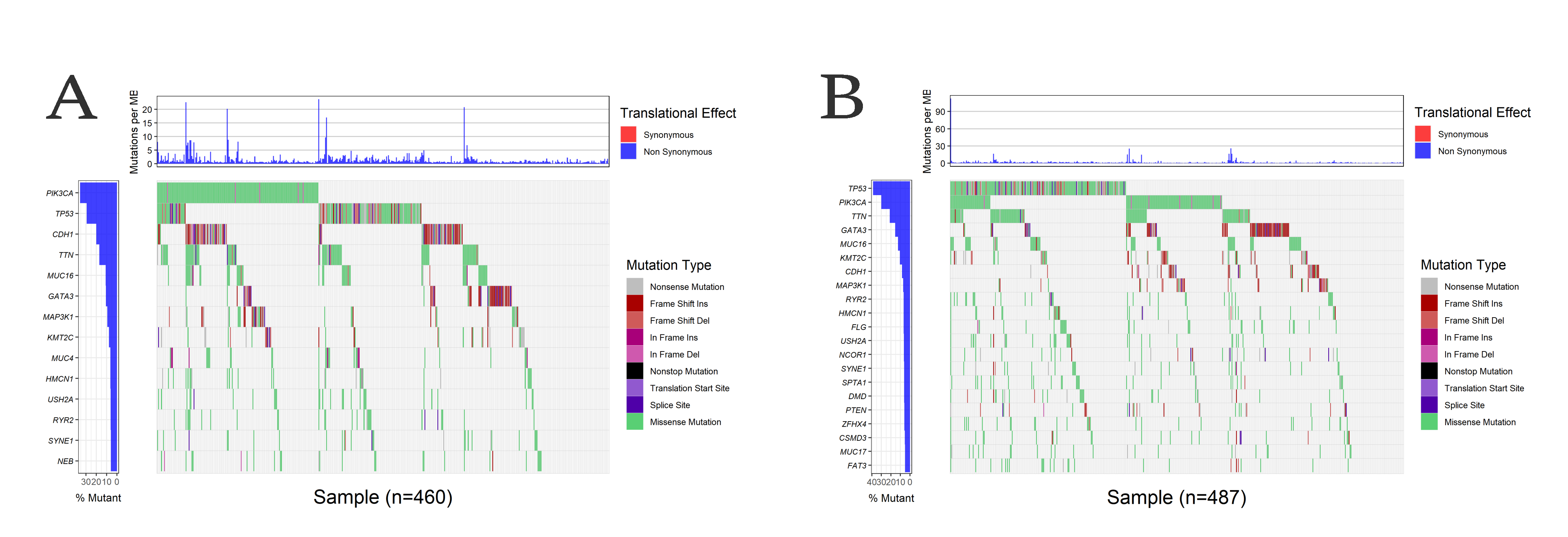

Supplement: Supplementary Figure 1 — Prognostic analysis of the 12-gene model in the TCGA datasets (A) The distribution of risk score in the TCGA datasets. (B) Kaplan-Meier survival analysis of OS between the risk group in the TCGA datasets. [file DataSheet_1.zip › Supplementary-/Supplementary Figure 3.tif]

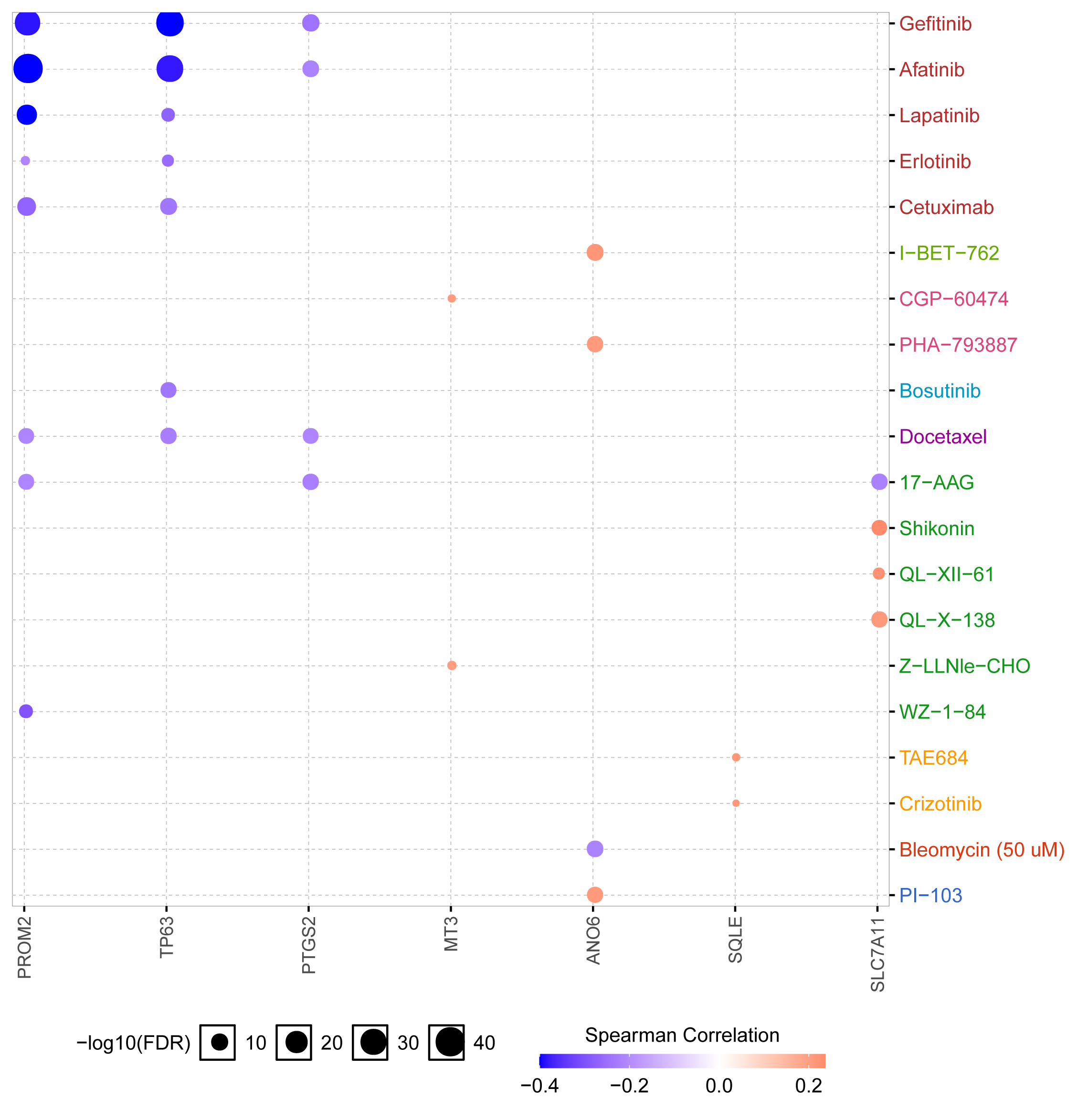

Supplement: Supplementary Figure 1 — Prognostic analysis of the 12-gene model in the TCGA datasets (A) The distribution of risk score in the TCGA datasets. (B) Kaplan-Meier survival analysis of OS between the risk group in the TCGA datasets. [file DataSheet_1.zip › Supplementary-/Supplementary Figure 4.tif]
